# Supplementary material for: Red Cusk-Eel (Genypterus chilensis) Gut Microbiota Description of Wild and Aquaculture Specimens
Source: Microorganisms. 2022 Jan 4;10(1):105. doi: 10.3390/microorganisms10010105 (PMC8779451; doi:10.3390/microorganisms10010105)
Supplement: Supplementary file 1 [file microorganisms-10-00105-s001.zip › microorganisms-1488463-supplementary.pdf]

microorganisms

Supplementary Material

Table S1. Summary of differences in pathways between predicted metagenomes.

	Wild	Aquaculture	P value
**Carbohydrate metabolism**			
Starch and sucrose metabolism	0,48 ± 0,09	**0,71 ± 0,02**	0,0048
Fructose and mannose metabolism	0,57 ± 0,06	**1,09 ± 0,06**	< 0,0001
Galactose metabolism	0,38 ± 0,08	**0,86 ± 0,05**	< 0,0001
Amino sugar and nucleotide sugar metabolism	0,53 ± 0,04	**0,80 ± 0,04**	< 0,0001
Pentose phosphate pathway	0,92 ± 0,11	**1,11 ± 0,04**	< 0,0001
**Lipid metabolism**			
Linoleic acid metabolism	**0,28 ± 0,06**	0,14 ± 0,02	0,0051
Arachidonic acid metabolism	**0,10 ± 0,02**	0,07 ± 0,00	0,0469
Fatty acid metabolism	**0,49 ± 0,10**	0,33 ± 0,05	0,0255
Fatty acid biosynthesis	0,88 ± 0,11	**1,06 ± 0,07**	0,0290
Synthesis and degradation of ketone bodies	0,42 ± 0,09	**0,55 ± 0,05**	0,0413
**Metabolism of cofactor and vitamins**			
Riboflavin metabolism	**0,56 ± 0,04**	0,37 ± 0,03	0,0002
Nicotinate and nicotinamide metabolism	**0,62 ± 0,05**	0,43 ± 0,03	0,0004
Biotin metabolism	**0,57 ± 0,06**	0,26 ± 0,02	< 0,0001
One carbon pool by folate	**0,92 ± 0,06**	0,84 ± 0,03	0,0374
Retinol metabolism	0,08 ± 0,01	**0,12 ± 0,01**	0,0007
**Metabolism of terpenoids and polyketides**			
Carotenoid biosynthesis	0,02 ± 0,02	**0,15 ± 0,01**	< 0,0001
Tetracycline biosynthesis	0,42 ± 0,05	**0,54 ± 0,04**	0,0066
**Xenobiotics biodegradation and metabolism**			
Dioxin degradation	0,17 ± 0,07	**0,41 ± 0,04**	0,0008
Polycyclic aromatic hydrocarbon degradation	0,11 ± 0,06	**0,27 ± 0,03**	0,0014

bold: indicates the condition (wild or aquaculture) when the value is higher.

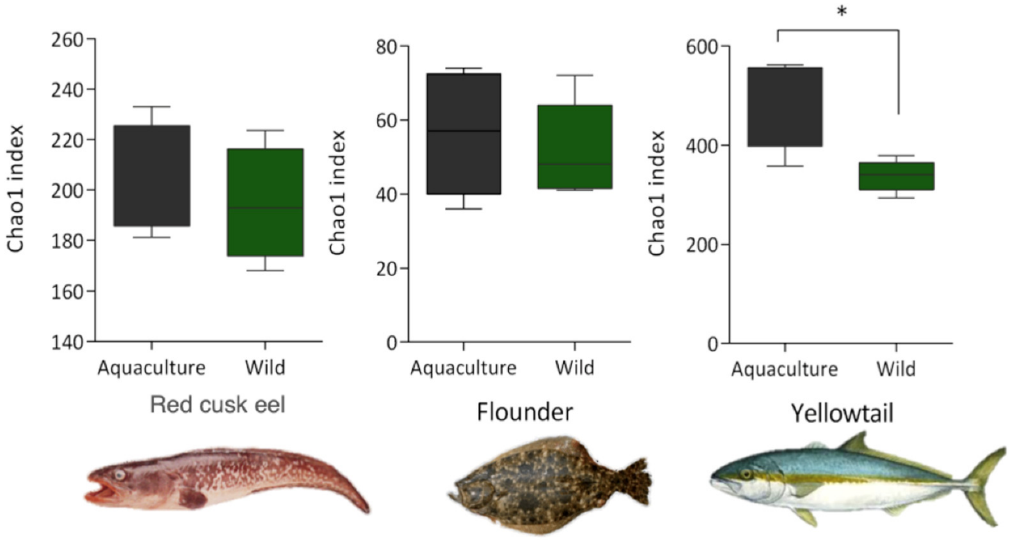

Figure S1. Alpha diversity comparison between wild and aquaculture in several Chilean fish. Diversity in the intestinal bacterial community was measured using Chao1 between wild (green) and aquaculture (gray).* indicates significant difference.

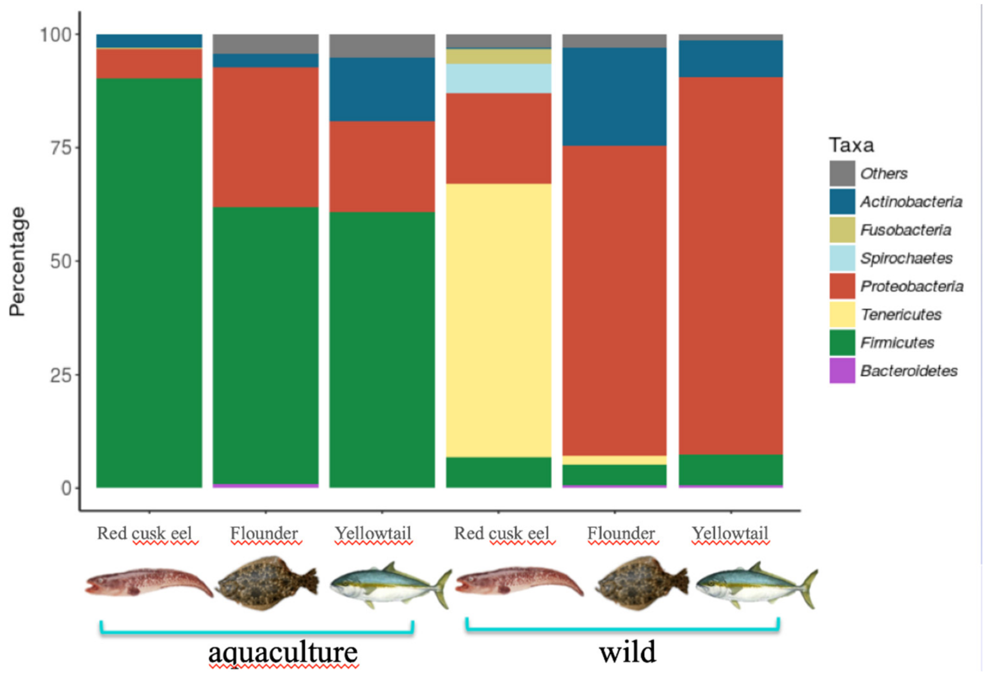

Figure S2. Comparison of microbiota composition between wild and aquaculture in several Chilean fish at phylum level. Average of relative abundance of each phylum was included for Red cusk eel, flounder and yellowtail, reared or captured in Chile.

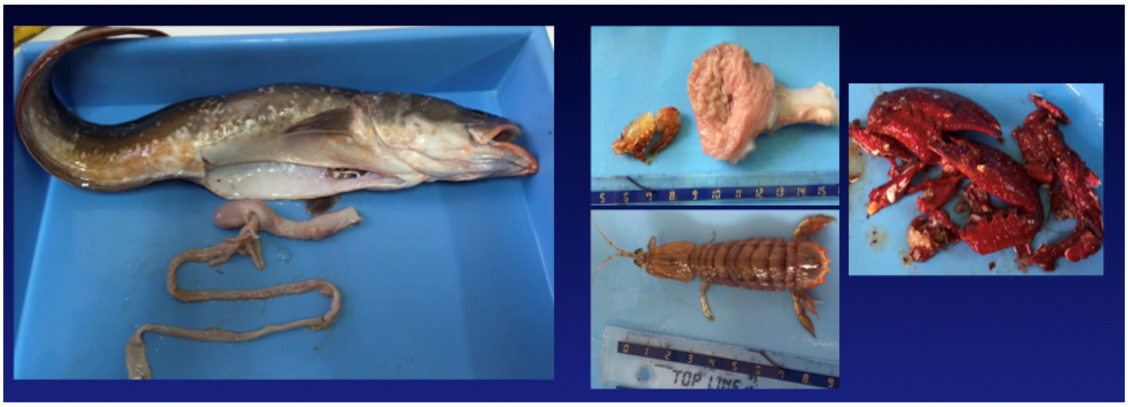

Figure S3. Gastrointestinal tract of *Genypterus chilensis* and the preys found in the stomach of several wild individuals.

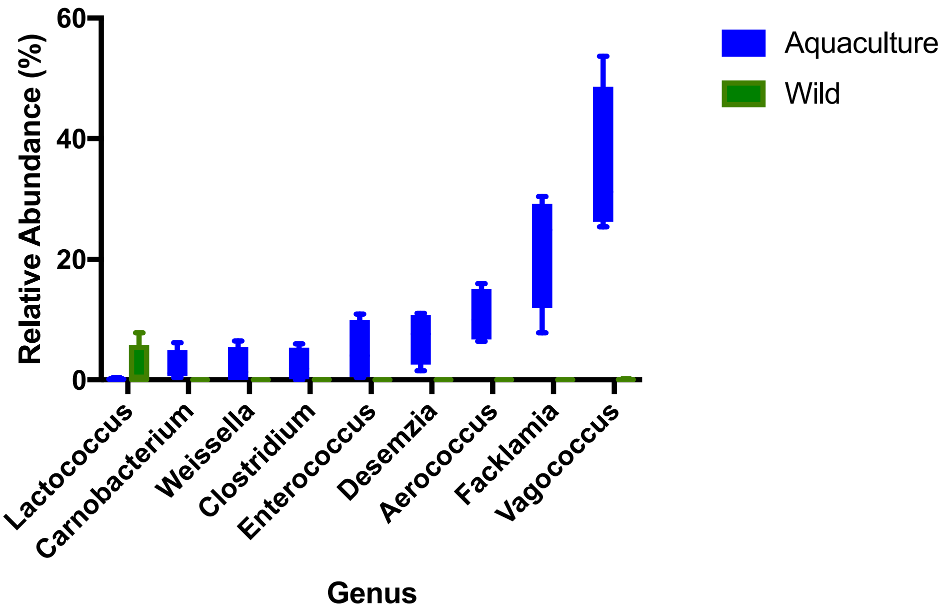

Figure S4. Relative abundance of genera belonging to *Firmicutes* found in intestinal contents of wild (green) or reared (blue) *Genypterus chilensis*.
